# Improving dental epithelial junction on dental implants with bioengineered peptides

**DOI:** 10.3389/fbioe.2023.1165853

**Published:** 2023-06-20

**Authors:** Ivan V. Panayotov, Attila G. Végh, Marta Martin, Boyan Vladimirov, Christian Larroque, Csilla Gergely, Frédéric J. G. Cuisinier, Elias Estephan

**Affiliations:** ^1^ LBN, University Montpellier, Montpellier, France; ^2^ CSERD, CHU Montpellier, Montpellier, France; ^3^ Biological Research Centre, Institute of Biophysics, Eötvös Lóránd Research Network (ELKH), Szeged, Hungary; ^4^ L2C, University Montpellier, CNRS, Montpellier, France; ^5^ Department of Maxillofacial Surgery, Medical University of Plovdiv, Plovdiv, Bulgaria; ^6^ Department of Nephrology, CHU Montpellier, Hôpital Lapeyronie, IRMB, University of Montpellier, INSERM U1183, Montpellier, France; ^7^ Neuroscience Research Center, Faculty of Medical Sciences, Lebanese University, Beirut, Lebanon

**Keywords:** bioengineered peptide, phage display, implants, titanium surface functionalization, epithelial adhesion

## Abstract

**Introduction:** The functionalization of titanium (Ti) and titanium alloys (Ti6Al4V) implant surfaces via material-specific peptides influence host/biomaterial interaction. The impact of using peptides as molecular linkers between cells and implant material to improve keratinocyte adhesion is reported.

**Results:** The metal binding peptides (MBP-1, MBP-2) SVSVGMKPSPRP and WDPPTLKRPVSP were selected via phage display and combined with laminin-5 or E-cadherin epithelial cell specific peptides (CSP-1, CSP-2) to engineer four metal-cell specific peptides (MCSPs). Single-cell force spectroscopy and cell adhesion experiments were performed to select the most promising candidate. *In vivo* tests using the dental implant for rats showed that the selected bi functional peptide not only enabled stable cell adhesion on the trans-gingival part of the dental implant but also arrested the unwanted apical migration of epithelial cells.

**Conclusion:** The results demonstrated the outstanding performance of the bioengineered peptide in improving epithelial adhesion to Ti based implants and pointed towards promising new opportunities for applications in clinical practice.

## 1 Introduction

Pure titanium (Ti) (Brannemark et al, 1977) and titanium alloys (Ti6Al4V) (Katsikeris et al, 1987) have been the most successful and widespread metals used for dental and maxillofacial implants. Inflammation and destruction of soft and hard tissues surrounding dental implants are termed mucositis and peri-implantitis (Roos-Jansaker et al, 2003). Mucositis points out a bacteria-induced, reversible inflammatory process of the peri-implant soft tissue with reddening, swelling and bleeding on periodontal probing. Peri-implant mucositis can lead to peri-implantitis involving loss of marginal alveolar bone around a functioning oral implant (Zitzmann and Berglundh, 2008). In contrast to mucositis, peri-implantitis is a progressive and irreversible disease of implant-surrounding hard and soft tissues showing bone resorption, decreased osseointegration, increased pocket formation and abscesses (Smeets et al, 2014).

Peri-implantitis has a prevalence on the order of 10% of implants and 20% of patients 5–10 years after implant placement (Mombelli et al, 2012; Heitz-Mayfield and Mombelli, 2014). Peri-implant mucositis occurs in about 80% of patients and in about 50% of implant sites (Zitzmann and Berglundh, 2008). Several epidemiological and clinical (Heitz-Mayfield and Mombelli, 2014) studies have pointed out that untreated peri-implantitis may threaten general health by increasing the risk of cardiovascular diseases, preterm labor and pulmonary diseases (Pussinen et al, 2007). Cardiovascular diseases that occur in untreated periodontitis or peri-implantitis as a risk factor lead to mortality and disability (Mattila et al, 2005).

As oral mucosa is transfixed by the implant, the epithelial sealing has been identified as the critical factor to prevent peri-implant inflammation (Rompen et al, 2006). The adhesion of oral keratinocytes must be stable and resistant to external aggressors, including mechanical constraints and bacterial pathogens and toxins. Up to now, “platform switching” is the only clinically proven strategy for the prevention of peri-mucositis and peri-implantitits (Atieh et al, 2010; Baffone et al, 2012). This concept is based on the use of the trans-gingival part of the dental implant (implant abutment) with a smaller diameter than the intraosseous part of the implant which assures a sufficient dimension of peri-implant mucosa to control epithelial-conjunctival attachment (Atsuta et al, 2012; Cumbo et al, 2013).

Biomaterials surface modifications are usually performed to increase their biocompatibility and bioactivity. These modifications are habitually divided into two types: additive and subtractive method (Kazimierczak and Przekora, 2020). Additive surface modifications are represented by inorganics (Fu et al, 2020; Mumith et al, 2020; de Oliveira et al, 2021) and organics surface coatings which incorporates surface functionalization with different inorganic and organic molecules, proteins and peptides (Werner et al, 2009; Souza et al, 2019; Lallukka et al, 2022).

Titanium (Ti) and titanium alloy (Ti6Al4V) modification types (mechanical surface modifications, oxidative processes, sol-gel derived titania (TiO_2_) coatings and biofunctionalized surfaces) on nonkeratinized soft tissues were reviewed. It was shown that Ti implants with a roughness between 0.5 and 1.0 μm induce soft-tissue adhesion and that a fibroblast growth factor 2 apatite composite coating promoted soft-tissue attachment via Sharpey-like fibers (Zigterman et al, 2019). According to Panayotov et al, (2015), three main surface modification approaches have been used in order to improve the tissue-implant interface of Ti and Ti6Al4V: layer-by-layer deposition of polyelectrolyte films, phage display-selected surface binding peptides and self-assembled DNA monolayer systems. Van den Borre concludes that porous titanium coatings having large pores (>700 μm) support cell attachment and that nanostructured ceramic coatings are found to reduce the inflammatory response. In this latter review, a particular interest was conferred for biomolecule coatings so that a durable fixation of the implant can be ensured (Van den Borre et al, 2022). Anodization has gained special interest in surface modification, it was stated that the anodized titanium surface increases blood clot retention and nano-roughness, and aids osseointegration (Traini et al, 2018). Some reports have been published on combining specific peptides that can lead to osteo-integration and bioactivity (Yazici et al, 2013; Rodriguez et al, 2017). Bifunctional chimeric peptides were also designed in order to prevent bacterial biofilm formation. The antibacterial potential of these novel peptides was studied against a set of different bacterial strains (Yucesoy et al, 2015; Liu et al, 2016; Yazici et al, 2016; Geng et al, 2018; Zhang et al, 2018; Wisdom et al, 2019; Wisdom et al, 2020; Drexelius et al, 2023). In these later studies, bi-peptides were synthetized with two domains, the first one was for Titanium binding with a robust solid-surface coating and the second with antimicrobial properties. In order to promote bone regeneration, a bioinspired chimeric peptide was also designed to activate the canonical Wnt/β-catenin signaling pathway of stem cells (Zhou et al, 2015). This later peptide can enhance mineral deposition and osteogenesis. Zhao et al, (2021) used 3D printing technology and chimeric peptides in order to improve osseointegration on the implant−bone surface. According to Kumar Boda and Aparicio, (2022), a dual keratinocyte-attachment and an anti-inflammatory coating can help in reducing inflammation and promote permucosal/peri-implant soft tissue sealing. In this report, pristine and oxygen plasma pre-treated polished titanium was coated with conjugated linoleic acid and cationic cell adhesive peptides.

In the present investigation, we introduced metal-cell specific bifunctional peptides (MCSPs) designed to increase the gingival adhesion on the dental implant surface and inhibit the epithelial cell migration toward the apical part of the implant. This bifunctional peptide is a combined peptide composed of two parts (Brannemark et al, 1977): a specific peptide for the implant surface and (Katsikeris et al, 1987) a peptide with high affinity to endothelial cells. A set of physical and biological analyses was used to select the best candidates which were tested *in-vitro* and *in vivo* in a rat model.

## 2 Materials and methods

### 2.1 Substrate coating

#### 2.1.1 Substrate preparation

Ti and Ti6Al4V discs (d = 15 mm and thickness 1 mm) were polished on Silicon Carbide disks and on soft disks using diamond pastes (6 μm, 1 μm, and 0.25 µm) on a polishing machine (Escil, Lyon, France). Specimens were thoroughly cleaned in sodium dodecyl sulfate 0.1 M (Sigma Aldrich, St. Louis, United States), in hydrochloric acid 0.1% (Sigma Aldrich, St. Louis, United States) and finally in ultra-clean water (Milli-Q; Merck Millipore, Darmstadt, Germany) at 22°C in an ultrasound bath for 5 min. For cell incubation, all surfaces were cleaned in a 70% alcohol bath for 10 min. Finally, the samples were washed tree times and stored in PBS (Gibco^®^, Invitrogen, Carlsbad, CA, United States) under sterile conditions. Polishing and cleanup with the same protocol procedures were repeated before phage display cycles and before each manipulation. After polishing and cleaning the substrate roughness were verified using AFM. A roughness of about 10 nm was obtained for the two type of substrates.

#### 2.1.2 Phage display selection of metal binding peptides

An M13 bacteriophage library (PhD-12 PD Peptide Library Kit™) supplied by New England Biolabs (Beverly, MA, United States) in phosphate-buffered saline solution containing 0.1% TWEEN-20 (PBST) was exposed to Ti and Ti6Al4V samples. After rocking for 1 h at room temperature, the Ti and Ti-alloy surfaces were thoroughly washed with PBST to rinse off unbound phages. Bound phages were then eluted from the surface under acidic conditions (glycine-HCl pH 2.2, 10 min), which disrupt the interaction between the displayed peptide and the target. Before elution, target wells were changed to prevent the elution of phages bound to the plastic walls. After neutralization with Tris-HCl (pH 9.1), the eluted phages were infected into the bacterial host strain *Escherichia coli* ER2738 and thereby amplified. After three to six rounds of biopanning, monoclonal phage populations were selected and analyzed individually. Finally, ten phages were selected and amplified from each sample, followed by the extraction of their DNA to determine the genetic code of the expressed peptide.

### 2.2 Physicochemical characterization of the metal binding peptides

#### 2.2.1 MALDI-TOF/TOF analysis and substrate coating with MBP

Ti and Ti6Al4V surfaces were incubated in a 100-µM solution of SVSVGMKPSPRP peptide (MBP-1) or WDPPTLKRPVSP peptide (MBP-2) for 2 h. The samples were rinsed thoroughly, either with a hydrophilic solution: ultra clean water (Milli-Q; Merck Millipore, Darmstadt, Germany), a hydrophobic solution: acetonitrile 100% (Sigma—Aldrich St. Louis, MO, United States) or an ionic solution (NaCl, 1 M). The presence of the peptides on the dry surfaces was identified with MALDI TOF/TOF spectrometry. Samples were analyzed using a 4,800 Plus MALDI-tandem time-of-flight system (MALDI-TOF/TOF) Proteomics Analyzer (Applied Bio systems, Foster City, CA, United States) in positive reflector ion mode using a 20-kV acceleration voltage. The YAG laser was operated at a 200-Hz firing rate with a wavelength of 355 nm. Mass spectrometry spectra were acquired for each sample using 1,500 laser shots. All acquired spectra of the samples were processed using the 4,000 Series Explorer TM software (Applied Bio systems, Foster City, CA, United States) in default mode.

The peptide was identified by searching in the Swiss-Prot database using Protein Pilot TM 2.0 software (Applied Bio systems, Foster City, CA, United States) or Protein Prospector (http://prospector.ucsf.edu/). The ExPASy database (www.expasy.org/tools/pi_tool.html) was used to calculate the mono isotopic theoretical mass of the peptide.

#### 2.2.2 Atomic force microscopy

AFM measurements were performed using an Asylum MFP-3D head and controller (Asylum Research, Santa Barbara, CA, United States), mounted on an Olympus inverted microscope. Height images were recorded in tapping mode and in liquid at room temperature. Typically, 512 × 512 points scans were taken at a scan rate of 1 Hz per line. Both trace and retrace images were recorded and compared.

##### 2.2.2.1 Force measurements by atomic force microscopy

Relative binding strengths of peptides onto Ti and Ti6Al4V surfaces were measured in contact mode and in a liquid medium (PBS pH 7.4) with a functionalized tip. Force measurements were taken at constant loading rates (vertical piezo-velocity of 1 μm/s). The spring constant of the tip was calibrated in the presence of PBS solution using the thermal fluctuation method and found to be approximately 18 pN/nm. For tip functionalization, the ultrasoft AFM cantilever tips (Bio lever-Olympus) were rinsed with copious amounts of Milli-Q water and then dried. In the next step, tip functionalization was performed. The AFM cantilever tips were incubated in 1 μg/mL biotinylated bovine serum albumin (BSA; Sigma Aldrich, St. Louis, MO, United States) solution in PBST, pH 7.0, at room temperature overnight, and the tip was then incubated for 30 min in 100 μg mL^−1^ streptavidin in PBST and finally in BSA (1%) for 1 h to block the nonspecific binding sites. Thorough rinsing was performed between all steps. Biotinylated peptides were fixed on the tip prior to each measurement.

### 2.3 *In vitro* testing

#### 2.3.1 Bi-functional peptides

Four bi-functional metal binding cell specific peptides (MCSPs) were synthetized (MilleGen, Toulouse, France) with a purity higher than 80%. Titanium and Ti6Al4V surfaces were incubated in 100 µM PBS solutions of MCSPs for 2 h and were then washed three times with PBS (Invitrogen, Carlsbad, CA, United States) before cell incubation.

#### 2.3.2 Oral keratinocyte cells

Cells from a non-tumoural, immortalized oral keratinocyte cell line, TERT-2 OKF-6 (BWH Cell Culture and Microscopy Core, United States) were cultivated in defined keratinocyte serum-free medium (KSFM; Gibco^®^, Invitrogen, Carlsbad, CA, United States) supplemented with: CaCl_2_ (Sigma Aldrich, St. Louis, MO, United States), 0.25 µg Bovine Pituitary Extract (BPA), 0.2 ng/mL epithelial growth factor (EGF), and 0.3 mM, 10% Pen Strep X 100 (Penicillin–10,000 Unit/mL, Streptomycin–10,000 mg/mL) (Gibco^®^, Invitrogen, Carlsbad, CA, United States). The medium was changed every 2 days until cells were used. For all experiments, cells from passage number 7 to 9 were used. After reaching 90% confluence, the cells were detached with 0.05% Trypsin-EDTA (Gibco^®^, Invitrogen, Carlsbad, CA, United States) for 5 min.

#### 2.3.3 Atomic force microscopy for *in vitro* studies

Tip-less cantilevers (MikroMasch, Tallinn, Estonia) were used for single cell-implant surface adhesion measurements. Determination of the spring constant for each cantilever was performed by the thermal calibration method implemented in the driving software (Hutter et al, 1993; Sader et al, 1999), resulting in a value of 0.03 N/m. Individual oral keratinocyte cells were attached to surface activated tip-less cantilevers using the protocol of Zhang et al, (2006) (41) to obtain concanavalin-A (Con A) mediated linkage. All measurements were conducted with the cantilever and the attached cell immersed in complemented KSF medium (Gibco^®^, Invitrogen, Carlsbad, CA, United States) at 30°C and within 3 h, allowing comparison of the adhesion forces for types of surfaces with the same cell. The measurements were taken at five different points on each surface. Force measurements were performed with a loading rate of 2 μm/s and a load of 2 nN (see Supplementary Information).

#### 2.3.4 Para-nitrophenyl-phosphate (pNPP) cell viability test

In order to analyze the protein phosphatase activity of the cells, the pNPP assay was performed for each of the studied Ti and Ti6Al4V surfaces with and without peptide functionalization at 4 h after cell incubation. Confluent cells were washed with PBS and detached with Trypsin-EDTA 0.5% (Gibco^®^, Invitrogen, Carlsbad, CA, United States) for 5 min at 37°C. Cells were then re-suspended in supplemented KSFM, and 5 × 10^5^ cells per well were incubated with the bare and functionalized Ti and Ti6Al4V surfaces. The samples were incubated at 37°C under a 5% CO_2_ humidified atmosphere for 4 hours. At the end of the incubation, the cells were washed three times with PBS and lysed with 500 µL of the acid phosphatase lysing buffer (0.1 M sodium acetate, 0.1% Triton X-100, pH 5.5), supplemented with 1 mg/mL of pNPP (para-Nitrophenyl Phosphate, Sigma Aldrich, St. Louis, MO, United States). After 1 h incubation at 37°C, the reaction was stopped by the addition of 50 µL of 1 N NaOH for 30 min at room temperature. The yellow colorimetric reaction was measured with a micro titer plate reader (EL-800 Universal Micro plate Reader, Bio Tec Instruments INC., VT, United States) at 405 nm. A linear relationship between the percentage of adhering cells and the light absorption due to para-nitrophenyl phosphate coloration was used to determine the concentration of adherent cells.

### 2.4 *In vivo* testing

The study was approved by the committee for animal welfare of Montpellier University with referral number 1143 15/03/2015.

#### 2.4.1 Oral implantation

The oral implantation procedure was completed according to the immediate-implantation protocol, as described by (Ikeda et al 2000) (42). Eighteen 12-week-old Wistar rats (male, 300–320 g) were anesthetized with intra peritoneal pentobarbital sodium (50 mg/kg; ref), and the first right maxillary molar was extracted. Ti6Al4V transgingival implants were immediately implanted after extraction for 4 weeks period. Bare implant surface, MCSP-2 functionalized surface and MBP-1 functionalized surface (6 implants/group) were compared. (see Supplementary Information).

#### 2.4.2 Tissue preparation

After 4 weeks, the rats from three groups were sacrificed and the samples were withdrawn, followed by 4% paraformaldehyde (Ph 7.4 in PBS) incubation, at 4°C for 4 h. Samples were demineralized in 5% EDTA, 4% sucrose in 0.01 m PB, Ph 7.4, for 4 days at 4°C. The oral mucosa surrounding the implant was then carefully removed from the implant. The gingiva around the left first molar was also removed from the tooth. All specimens were immersed in 20% sucrose in 0.1 m PBS at 4°C overnight for cryoprotection, and then embedded in O.C.T. compound. They were then quickly frozen in dry ice/isopentane, before cutting 10-mm bucco-palatal sections with a cryostat at 20°C. The cryo-sections were mounted on gelatin-coated glass slides.

#### 2.4.3 Immunohistochemistry

Laminin-332 γ2 IHC was using the avidin-biotinylated peroxidase complex (ABC) kit (Vector Laboratories, Burlingame, CA, United States) was performed. After sectioning the samples were washed in 0.01 M phosphate-buffered saline (PBS), pH 7.2, treated with 0.3% H_2_O_2_ for 30 min to inhibit endogenous peroxidase activity, and blocked for 30 min with 10% normal goat serum in PBS. The tissue sections were then incubated overnight at 4°C with affinity-purified polyclonal rabbit IgG antibody to rat γ2 chain of laminin-332 in PBS (1:100 or 1:50) for 48 h. The samples were then rinsed and incubated for 45 min with biotinylated goat anti-rabbit IgG in PBS (1:200), followed by a 60-min incubation with ABC dissolved in PBS (1:100). Immuno-positive staining was visualized by incubating the samples for 5 min in 0.02% diaminobenzidine-tetrahydrochloride (ABC kit) and then counterstaining them lightly with hematoxylin. The length of immunohistochemical coloration of laminin 332 indicate the epithelial adhesion on the implant surface was measured. This was observed like a dark-brown line at the implant-epithelium interface. All samples were observed with a light microscope Zeiss Axiolab comporting Zeiss F40/0.65 dry objectives (Carl Zeiss Industrielle Messtechnik GmbH, Oberkochen, Germany) and photographed with a Sony α5100 camera (Sony corporation, 1-7-1 Konan Minato-ku, Tokyo, 108-0075 Japan). All measurements were made using ImageJ software (NIH, Bethesda, MD, United States, http://rsb.info.nih.gov/ij/), after image calibration.

### 2.5 Statistical analysis

Data analysis was performed using Stata software v14.2 (StataCorp LP 4905 Lake way Drive College Station, Texas 77845-4512. United States). Details on the statistical analysis are provided below separately for the different studies performed.

#### 2.5.1 *In vitro* studies

##### 2.5.1.1 Single-cell force spectroscopy (SCFS) assay

Sample size calculation: SCFS measurement was performed at five different points of each of the studied surfaces. A minimum of 30 measurements were required to underline a minimal difference of 1 × 10^−9^N between groups, with a standard deviation of 1.5 × 10^−9^N and a power of 0.9.

The normality of each group of measurements was checked for the five different points by the Shapiro-Wilks test. For each of the five groups, the Gaussian distribution could not be rejected:

For titanium (Ti) surfaces:

Ti (*p* = 0.10),

MCSP-1 (*p* = 0.07),

MCSP-2 (*p* = 0.18), MCSP-3 (*p* = 0.06), MCSP-4 (*p* = 0.47).

For titanium alloy (Ti6Al4V) surfaces: Ti6Al4V (*p* = 0.46), MCSP-1 (*p* = 0.17), MCSP-2 (*p* = 0.06), MCSP-3 (*p* = 0.11), MCSP-4 (*p* = 0.83).

During the BSA inhibition essay: Ti (*p* = 0.06), Ti6Al4V (*p* = 0.43), Ti/MCSP-2 (*p* = 0.49), Ti6Al4V/MCSP-2 (*p* = 0.09), Ti/MCSP-2+P2+BSA (*p* = 0.58), Ti6Al4V/MCSP-2+BSA (*p* = 0.99).

Statistical tests: ANOVA with pairwise comparisons between groups was performed, considering the Bonferroni correction. A *p*-value of < 0.05 was considered as significant.

##### 2.5.1.2 Para-nitrophenyl-phosphate (pNPP) assay

The sample size was computed for testing the row effect for a 5%-level test with 90% power. With an effect variance of 0.2, the number of measures per group was at minimum 9. Since the Shapiro-Wilks normality test showed that the data were not normally distributed, a root square transformation was applied to the data to obtain a Gaussian distribution Ti (*p* = 0.15), Ti6AL4V (*p* = 0.35). A two-way ANOVA was used to test the two main effects: material and peptide on the adhesion forces. A *p*-value of < 0.05 was considered as significant.

#### 2.5.2 *In vivo* study

Sample size calculation: since repeated measures were performed within the same animal, a cluster effect was considered. With a difference in epithelial height of 120 μm, a standard deviation of 80 μm, an intra class correlation coefficient of 0.03 and a power of 80%, the number of rats required was calculated to be equal at least to 6 per group, with 4 measurements per rat and a one-sided test (α risk of 5%).

Normality was checked for the three groups by the Shapiro-Wilks test: bare Ti (*p* = 0.14), SVSVGMKPSPRP (MBP-1) coated Ti (*p* = 0.10) and WDPPTLKRPVSP (MBP-2) coated Ti (*p* = 0.76).

Statistical tests: One-way ANOVA with pairwise comparisons between groups was performed, with a Bonferroni correction.

## 3 Results

### 3.1 Selection and affinity of metal binding peptides (MBP)

**FIGURE 1:**
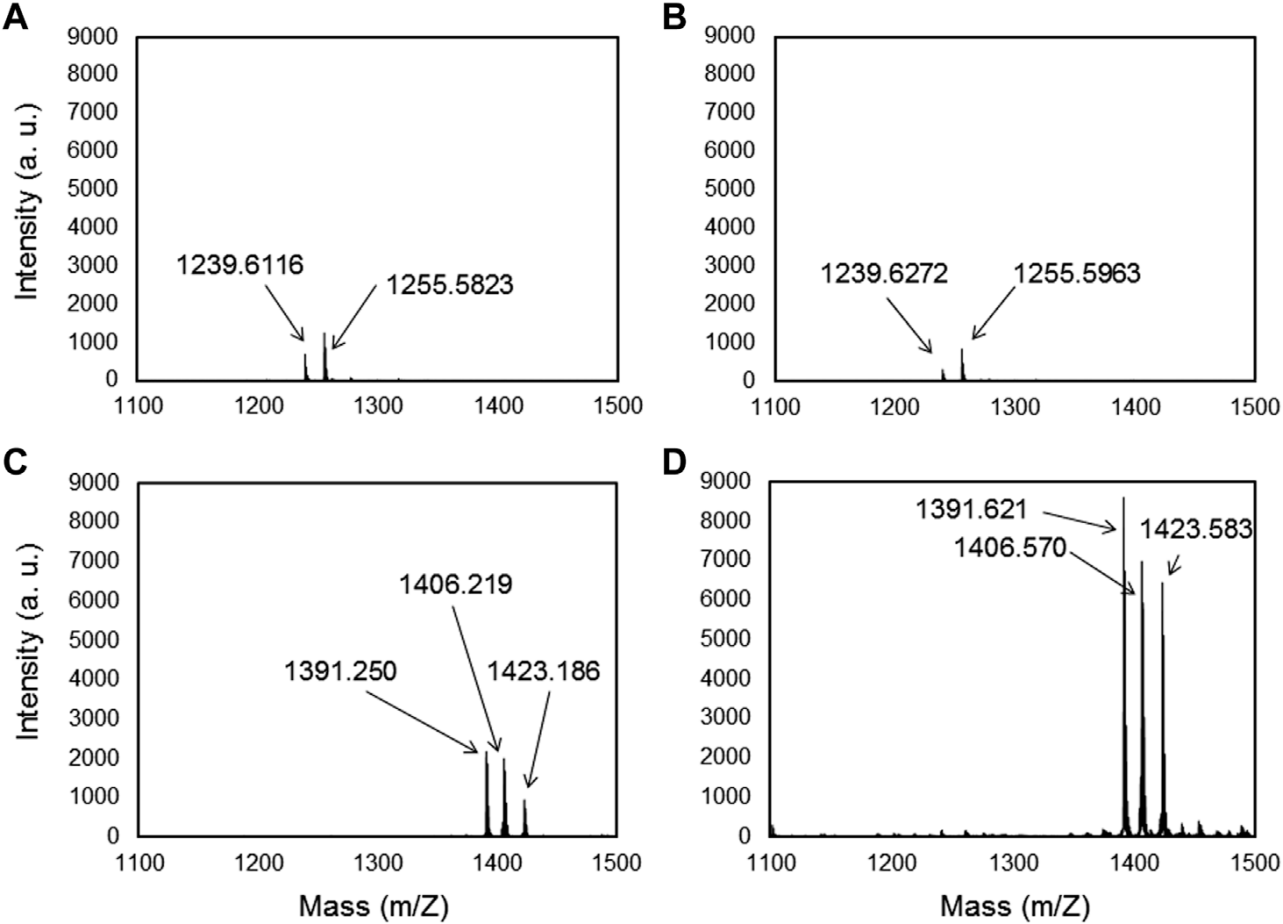
Maldi-TOF/TOF spectra of MBP-1 **(A,B)** and MBP-2 **(C,D)** on Titanium and Ti-alloy (Ti6Al4V) surfaces, respectively, after acetonitrile rinsing.

The metal binding peptides were selected via phage display using the M13 bacteriophage library and by performing four biopanning rounds against Ti6Al4V. SVSVGMKPSPRP (MBP-1) and WDPPTLKRPVSP (MBP-2) were selected as two specific peptides for our materials. MBP-1 was expressed by 37.5% of the phages after the third round of bio panning while MBP-2 was manifested at a rate of 40% after the fourth round (see Supplementary Information). The affinity of both MBPs to Ti and Ti6Al4V was tested by mass spectrometry and force spectroscopy. Concerning the mass spectrometry after adsorption of MBP-1 and MBP-2 on the Ti and Ti- alloys substrate, the samples were rinsed with acetonitrile. Figure 1 shows that the MBPs remain at the surfaces. Thus, these selected peptides present a high affinity to our substrates. The obtained mass-to-charge ratio (m/z) for MBP-1 was 1,239.6, in agreement with the theoretical mass of the peptide, and on both metals, we identified a small peak at an m/z of 1,255.6, corresponding to the oxidized form of the peptide (Figure 1A for MBP-1-Ti and Figure 1B for MBP-1-Ti- alloy). The obtained mass-to-charge ratio (m/z) for MBP-2 was 1,391.6, which corresponds to the theoretical mass of the protonated molecule [MH]+. Mass spectroscopy also reveals two other peaks at m/z 1,406.6 and m/z 1,423.6 suggesting the presence of oxygen ions in the peptide structure (Figure 1C for MBP-2-Ti and Figure 1D for MBP-2-Ti- alloy). The slight differences in the obtained peptide masses on the different materials were due to variations in the thickness of the samples, which induced small differences in the time of flight (see Supplementary Information).

**FIGURE 2:**
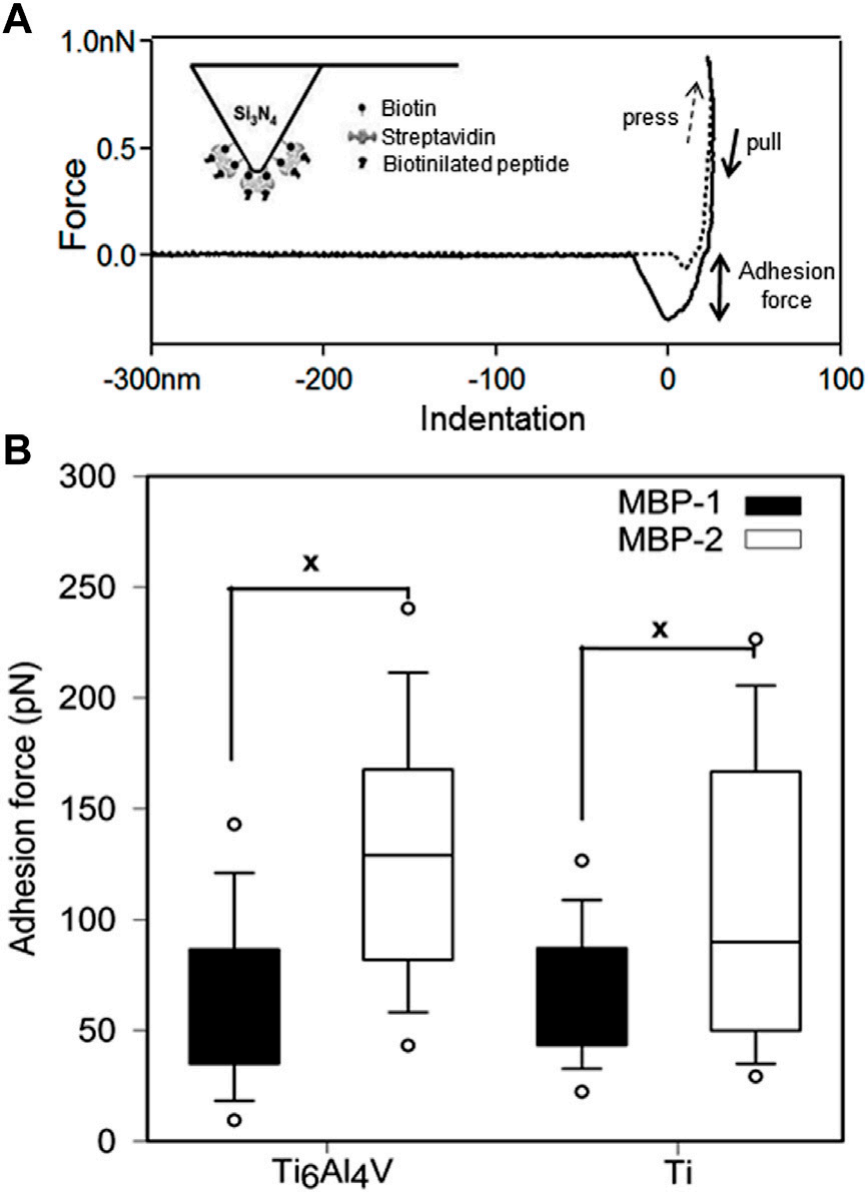
Force spectroscopy of metal binding peptides on Ti and Ti6Al4V. **(A)** Typical adhesion force curve measured with AFM. The inset shows a schematic representation of the peptide-functionalized tip. **(B)** Adhesion forces of MBP-1 and MBP-2. The MBP-1 adhesion forces on Ti6Al4V and Ti are 67.07 ± 1.34 pN and 65.42 ± 2.48 pN, respectively. The MBP-2 adhesion forces on the two surfaces are 109.76 ± 2.62 pN and 134.61 ± 2.65 pN, respectively.

To have a clearer view of the affinity of MBP-1 and MBP-2 to the metals, we conducted force spectroscopy using AFM. Monitoring the unbinding processes of adsorbed molecules under external stress allows the quantification of adhesion forces (Gergely et al, 2000; Gergely et al, 2002). The adhesion forces measured between MBPs and Ti and Ti6Al4V surfaces are presented in Figure 2. The MBP-2 peptide exhibited statistically significantly higher adhesion forces to both metals. The adhesion of MBP-2 on Ti6Al4V was almost two-fold stronger than the adhesion force of MBP-1. For comparison, the strength of the osteopontin and αvβ3 integrin bond is 50 ± 2 pN (Litvinov et al, 2003), that of the ICAM-1 and Anti-ICAM-1 antibody bond is 100 ± 50 pN (Willemsen et al, 1998), and cadherin-mediated cell-cell interactions have a minimal binding force in the range of 50 pN (Panorchan et al, 2006; Sivasankar et al, 2009). The highly specific biotin-streptavidin interaction is the strongest non-covalent bond, ranging from 250 to 320 pN, according to the experimental conditions (Wong et al, 1999). The interactions of MBP-1 and MBP-2 with the two metal surfaces are in the range of antigen-antibody forces.

### 3.2 Design of bi-functional peptides for epithelial cell attachment to titanium surfaces

**FIGURE 3:**
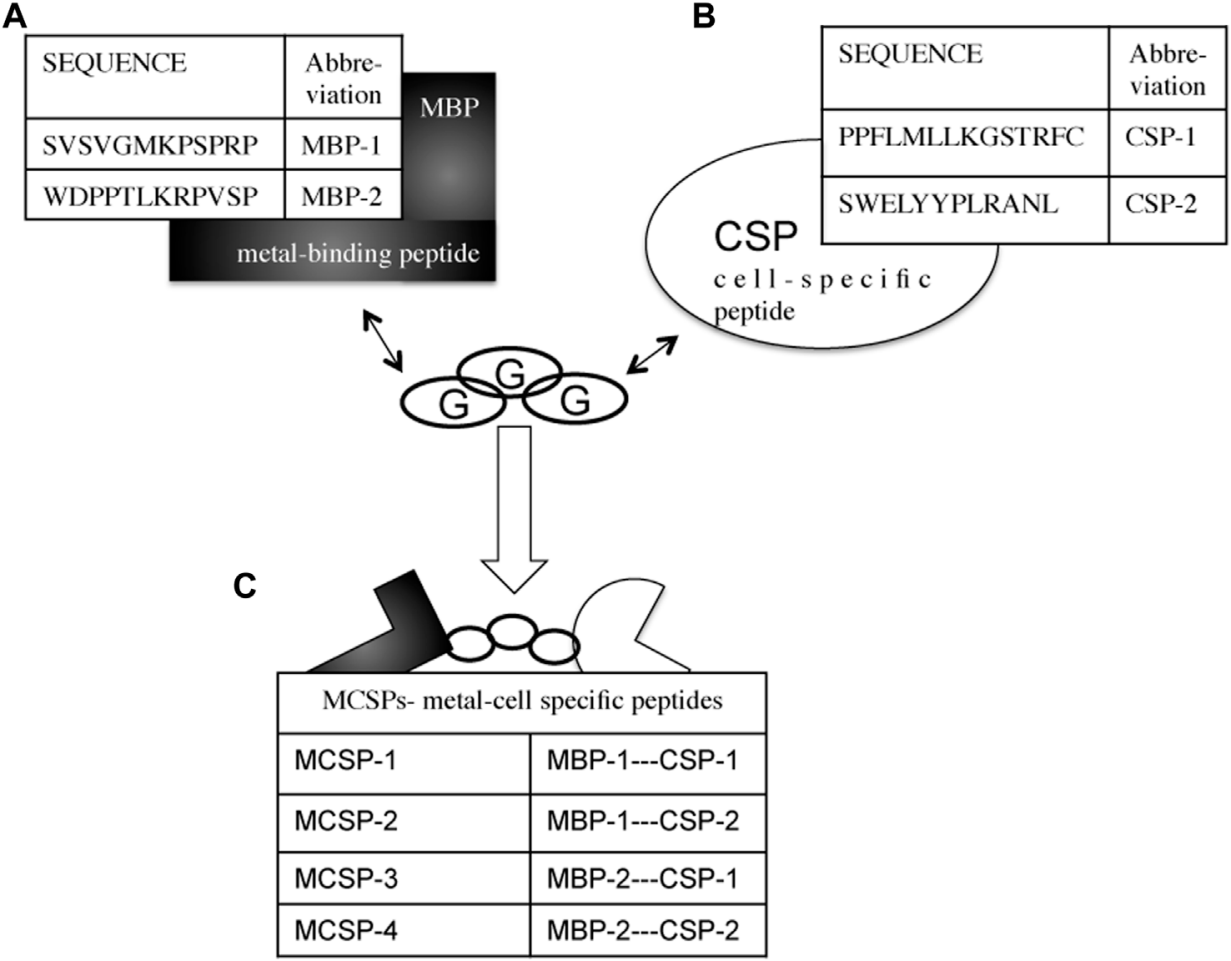
Combinational design of metal-cell specific peptides (MCSP). **(A)** Metal-binding peptides (MBPs) with high affinity to Ti and Ti6Al4V. **(B)** Cell specific peptides (CSPs) with affinity to laminin-332 (CSP-1) and to E-cadherin’s ectodomains (CSP-2). **(C)** The engineered metal-cell specific peptides (MCSPs). Three glycines (G) residues are used as a spacer between the two peptide sequences.

Four bifunctional, metal-cell specific peptides (MCSPs) were engineered by combining a 12-mer metal binding peptide (MBP-1, MBP-2) to a cell specific sequence (CSP-1, CSP-2) (Figure 3). Both MBPs were selected by phage display on titanium alloy surfaces (see Supplementary Information). The CSP-1 was selected because of its affinity for the laminin-LG3 globular domain (14 residues) (Kim et al, 2005; Werner et al, 2009), whereas the CSP-2 for its affinity to N- and E-cadherin ectodomains (12 residues) (Devemy and Blaschuk, 2009). These two types of cell receptors are found in the major classes of epithelial extracellular cell adhesion proteins (Smeets et al, 2014). The four MCSPs were composed of 29 or 27 residues, depending on the length of the CSP, including a spacer of three glycine amino acids between the metal binding peptide and the cell-specific peptide (Figure 3).

### 3.3 *In vitro* cell adhesion studies on Ti and Ti6Al4V alloy modified with engineered metal-cell specific peptides

**FIGURE 4:**
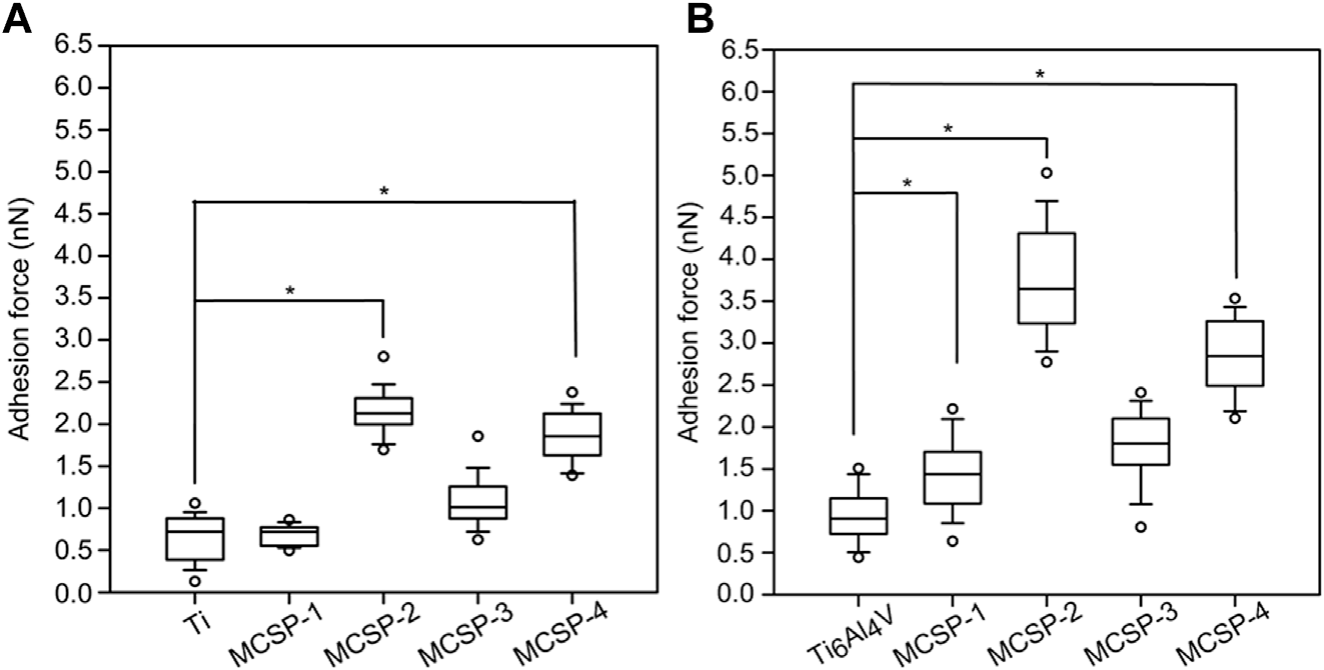
Strength of keratinocyte cell binding to bare and modified Ti and Ti6Al4V surfaces. Adhesion forces between living keratinocytes and metal surfaces were evaluated using AFM in force mode. **(A)** Akeratinocyte-decorated cantilever was used to measure a set of forces on bare and functionalized Ti and **(B)** Ti6Al4V surfaces, respectively. Ti, Ti6Al4V, MCSP-1, MCSP-2, MCSP-3, and MCSP-4 correspond respectively to the bare surfaces and those functionalized with MCSP-1, MCSP-2, MCSP-3, and MCSP-4. Error bars for **(A,B)** represent the standard deviation of multiple experiments. *: Significant differences correspond to *p* = 0.0001.

Single-cell force spectroscopy was applied to evaluate the ability of the M-CSPs to increase oral keratinocyte adhesion to Ti and Ti6Al4V. Oral keratinocyte cells were attached to tip-less cantilevers by means of a concanavalin-A mediated linkage (Zhang et al, 2006; Vegh et al, 2012). During the recorded force curves a dwell period of 5 s was introduced, when the cell and the substrate were in contact. This short period implies that no information was recorded about how the cell responds to the surface on a longer time scale, thus excluding the effects of, e.g., changes in protein expression (Weder et al, 2009) and limiting our study to the study of mechanical properties of the cell recording the effect of the proteins and protein complexes that were already present at the cell membrane. To address the efficacy of the surface modification cell adhesion forces were measured against the bare Ti and Ti6Al4V surfaces, and after their functionalization with the MCSPs (Figure 4). Surfaces coated with MCSP-2 and MCSP-4 exhibited higher cell adhesion forces than surfaces functionalized with MCSP-1 or MCSP-3. A significant difference in cell adhesions to MCSP-2- and MCSP-4-functionalized Ti (of about 2 nN) compared to the non-functionalized Ti surface (0.8 nN) has been found. The MCSP-1 and MCSP-3 peptides incorporate a common cell-specific peptide motif (CSP-2) with different metal binding part (MBP) (Figure 3), but they did show lower affinity in terms of adhesion force compared to MCSP-2 and MCSP-4. Combining MBP-1 and CPS-2 (cadherin binding peptide) in MCSP-2 appears to be the most successful peptide configuration both for Ti and Ti6Al4V functionalization to promote oral epithelial cell adhesion. The obtained keratinocyte cell adhesion forces are in the range of typical cell-surface interactions reported in the literature (Puech et al, 2005; Taubenberger et al, 2007; Taubenberger et al, 2010)–(Puech et al, 2005; Taubenberger et al, 2007; Taubenberger et al, 2010).

**FIGURE 5:**
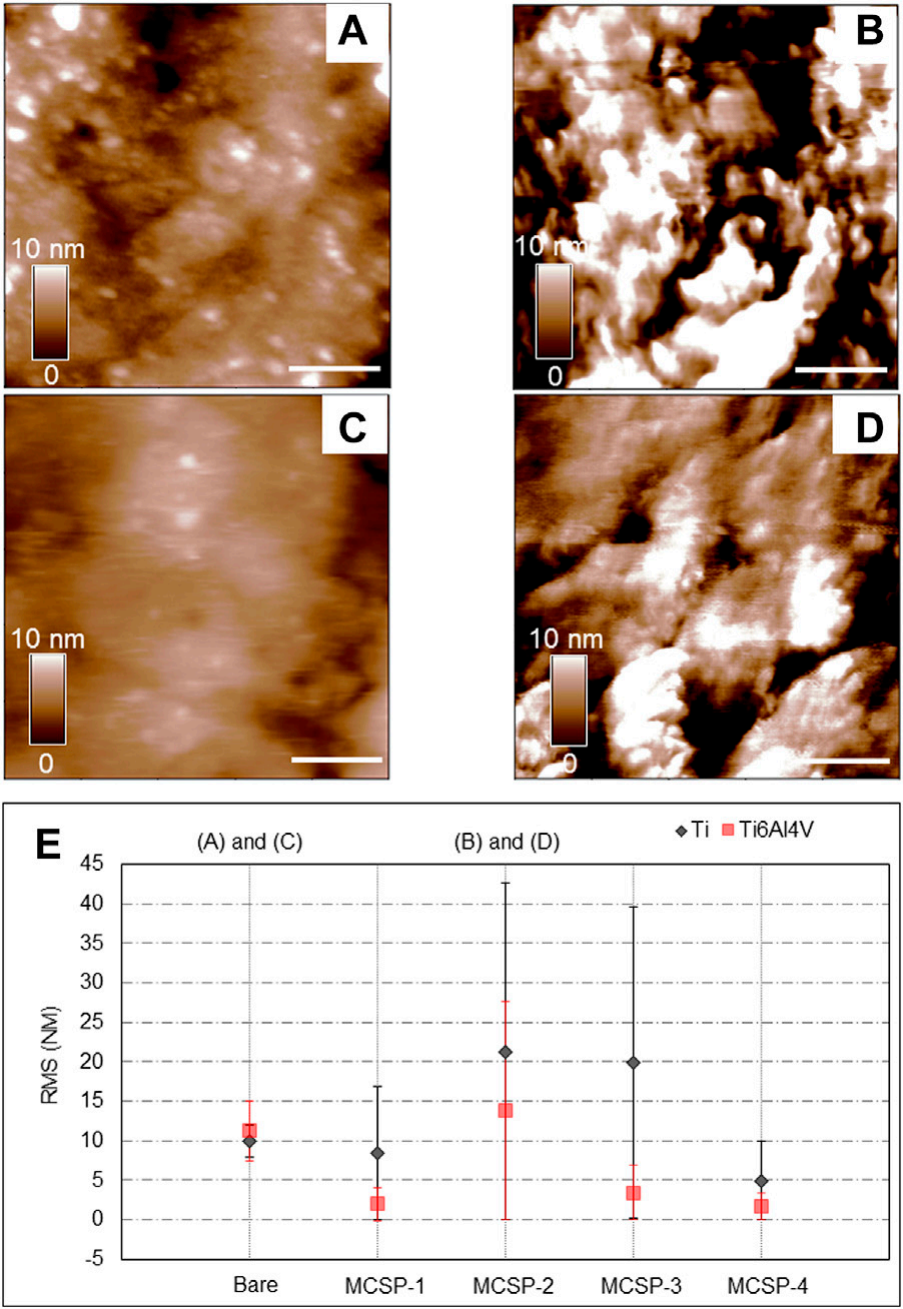
Evaluation of surface modification using AFM height images and surface roughness analysis. AFM height images (1 μm × 1 µm) were recorded in contact mode in liquid at 1 Hz. Height images for the bare and the MCSP-2 modified surfaces are presented: **(A)** Bare Ti surface, **(B)** Ti surface modified with MCSP-2, **(C)** Bare Ti6Al4V surface and **(D)** Ti6Al4V surface modified with MCSP-2. The scale bar on the height images **(A–D)** is 200 nm in length. The surface roughness of all measured surfaces is presented in graph **(E)**. The black data corresponds to the bare Ti and its four different coatings with MCSP-1, MCSP-2, MCSP-3, and MCSP-4. The red data correspond to the bare Ti6Al4V and its four different functionalized surfaces. The standard deviations of the surface roughness variations on the scanned surfaces are also shown.

The surface roughness is a crucial factor that influences cell attachment and proliferation on implants (Baharloo et al, 2005). Therefore, atomic force microscopy (AFM) was used to investigate the topography of the Ti and Ti alloy modified with the engineered metal-cell specific peptides. The surface roughness was characterized by calculating the root-mean-square (RMS) of each recorded topography. Figure 5 shows the obtained topographies for bare Ti (Figure 5A) and Ti6Al4V (Figure 5C) surfaces, and surfaces coated with MCSP2, where the highest RMS modifications were observed (Figures 5B, D, respectively). When modified with MCSP2 the surface roughness of bare Ti increases to about 21 nm from 9.6 nm (Figure 5E). Almost the same RMS increase was observed for the MCSP-3 functionalized Ti surface (Figure 5E). The RMS of a bare Ti6Al4V surface practically did not change after the adsorption of MCSP2. The AFM height images for both metal surfaces after functionalization with MCSP-2 demonstrated similar peptide deposition patterns with an agglomerates size of approximately 200 nm. This led to an increased variation of surface roughness after functionalization with MCSP-2 as indicated by the high values of RMS standard deviation. Functionalization with MCSP-1 and MCSP-4 did not increase the surface roughness of both metals (Figure 5E), however, the presence of MCSP-4 increased significantly cell adhesion on the surfaces (Figure 4). These results indicate that the higher cell adhesion forces measured for the MCSP-2 and MCSP-4 functionalized Ti and Ti6Al4V surfaces (Figure 4) cannot be explained solely by changes in the surface roughness.

**FIGURE 6:**
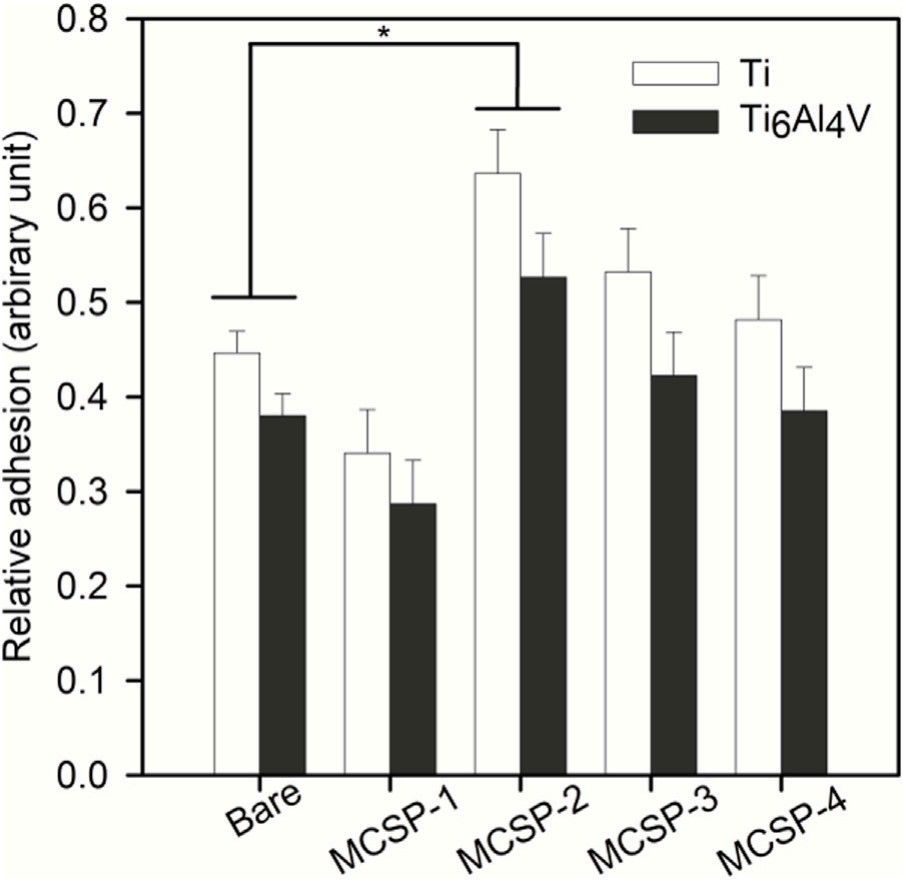
Assessment of oral keratinocytes adhesion in cell culture against bare and functionalized Ti and Ti6Al4V surfacesusing para-nitrophenil phosphate (pNPP) viability test. White bars correspond to cell adhesion on bare and functionalized (with MCSP-1, MCSP-2, MCSP-3, and MCSP-4, respectively) Ti surfaces, while black bars correspond to cell adhesion on bare and functionalized Ti6Al4V surfaces. * Significant differences (*p* = 0.0001).

**FIGURE 7:**
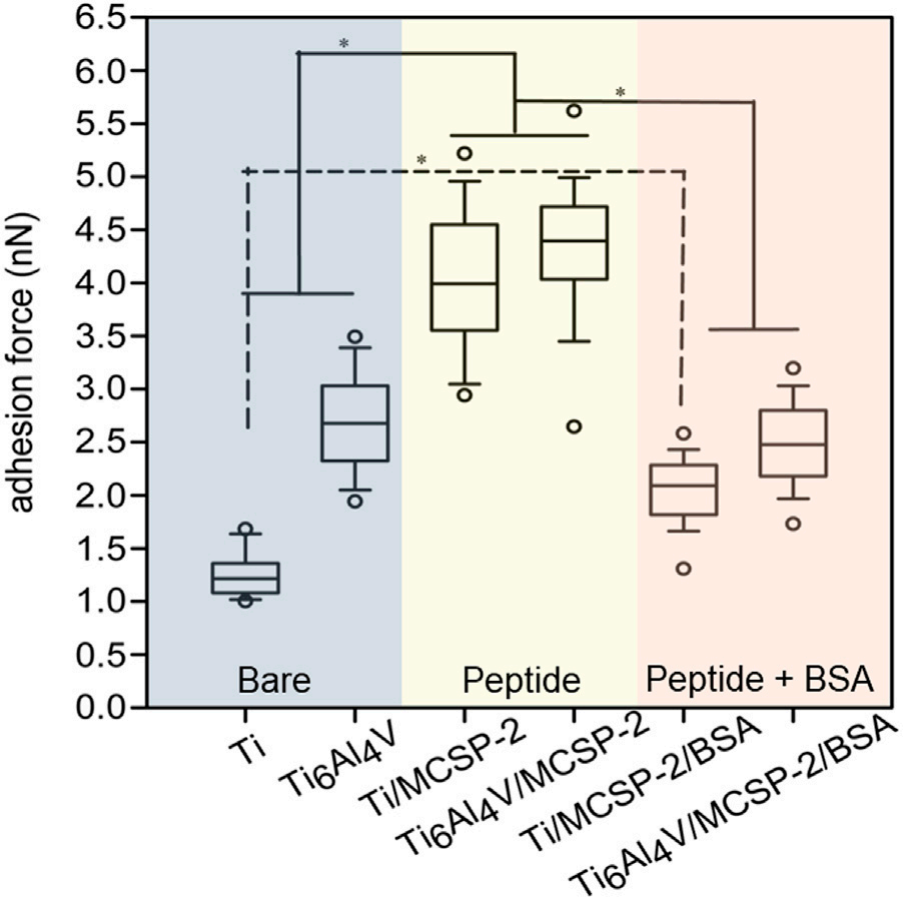
Cell adhesion after bovine serum albumin adsorption on metal-cell specific peptide-2. AFM was used in force mode to evaluate unbinding forces between cell functionalized tip-less cantilevers and three different surfaces of Ti and Ti6Al4V: the blue part corresponds to the bare Ti and Ti6Al4V surfaces, the yellow one shows data for Ti and Ti6Al4V functionalized with the MCSP-2, while the purple one corresponds to the Ti and Ti6Al4V functionalized with MCSP-2 and followed by BSA adsorption. The presence of BSA decreases the adhesion force to values measured for non-functionalized surfaces. Significantly higher cell adhesion was found against Ti/MCSP-2/BSA surfaces compared to bare Ti [*: Significant differences (*p* = 0.0001)]. Error bars represent the standard deviation of multiple experiments.

The cell adhesion measurements were completed with an *in vitro* para-nitrophenyl-phosphate (pNPP) cell viability test monitoring the adhesion of a high number of cells on the whole surface of the sample (Humphries, 2001). The number of adherent live cells was counted on functionalized and non-functionalized Ti and Ti6Al4V surfaces after 4-h incubation and elimination of non-adherent cells by rinsing. The results indicated a higher number of adherent cells on Ti surfaces (with or without functionalization) than on Ti6Al4V surfaces (Figure 6). Surfaces functionalized with MCSP-2 demonstrated a higher concentration of adherent cells which is statistically significant compared to the non-functionalized surfaces. The pNPP test revealed that MCSP-2 is the most effective bifunctional peptide for Ti and Ti6Al4Vsurface functionalization in view of increased oral keratinocyte adhesion. To test the robustness of the effect of MCSP-2, single-cell adhesion forces were compared before and after bovine serum albumin (BSA) adsorption on functionalized MCSP-2 surfaces. BSA is a widely used blocking agent to prevent nonspecific binding by inhibiting hydrophobic, ionic or electrostatic interactions (Gergely et al, 2004; Buchwalow et al, 2011) between proteins. Adhesion forces of a living oral keratinocyte cell against naked and MCSP-2-functionalized Ti and Ti6Al4V surfaces before and after BSA treatment were measured using AFM force spectroscopy (Figure 7). A statistically significant increase in the adhesion forces of living oral keratinocytes on MCSP2 functionalized Ti and Ti6Al4V surfaces was found compared to those measured against the bare surfaces. After BSA adsorption, cell adhesion to Ti6Al4V/MCSP-2 surfaces decreased drastically to the level of adhesion measured on the bare Ti6Al4Vsurface. Although cell adhesion forces decreased after BSA treatment in the case of Ti/MCSP-2 surfaces as well, they remained significantly higher than the adhesion measured against bare Ti.

### 3.4 *In vivo* study in a rat model

Finally, the capacity of MCSP-2 peptide to increase the epithelial cell attachment on implant (Ti6Al4V) surfaces *in vivo* was evaluated. Only one of the two *in vitro* tested surfaces was selected, because the Ti6Al4V alloy is the most commonly used material for trans-gingival implant abutments (Ritz et al, 2017). We expected difference in cell adhesion on functionalized and bare implants could be detected 4 weeks after implantation in the rat mouth, when the apical migration of epithelial cells could be evaluated. It is known that 4 weeks after the implantation the peri-implant junctional epithelium migrates further apically and occupies 40% of the total interface between the implant and the soft connective tissue that is rich in collagen fiber and fibroblasts (Sculean et al, 2014). This is an unwanted phenomenon that could influence the primary stabilization of the implant.

**FIGURE 8:**
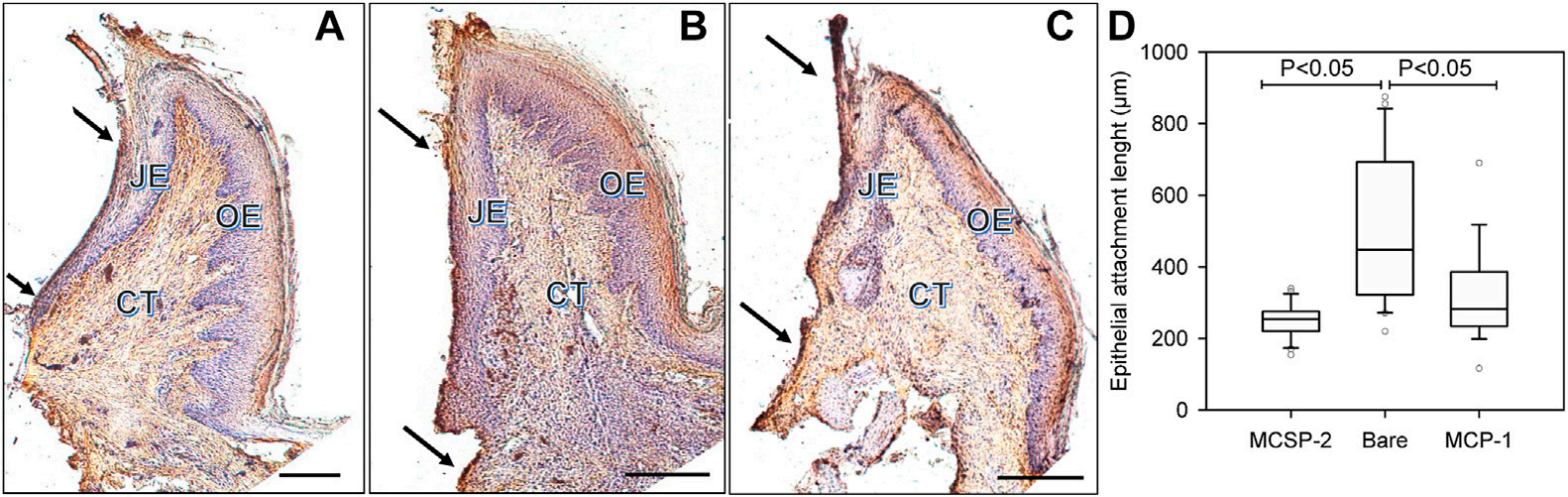
*In vivo* comparison of the epithelial adhesions on the peptide covered and bare implant surfaces, respectively. Histological slices of peri-implant gingiva in the fourth week after implantation are presented. The immunohistochemical coloration of laminin 332 indicates the epithelial adhesion on the implant surface. The dark arrows indicate cervical and apical points of measurement of the laminin 332 distribution, which is visualized like a dark brown line at the implant-epithelium interface; **(A)**: MCSP-2 covered Ti6Al4Vimplant; **(B)** bare implant; **(C)** MBP-1 covered implant; CT: connective tissue; OE: Oral Epithelium; JE: epithelial junction; Bare scale = 10 μm; **(D)** Statistical analysis of multiple measurements of the length of junctional epithelium for the 6 implanted animals. JE length after 4 weeks of healing demonstrates a statistically significant difference between the bi-functional peptides (MCSP-2) and the bare Ti6Al4V alloy (*p* ˂ 0.05). The length of JE indicating epithelial adhesion was smaller also for the MBP-1 coated implant, but the apical migration of the epithelial cells was not as limited as on the surfaces functionalized with MCSP-2.

Therefore, histological slices of the peri-implant gingiva interface were compared after 4 weeks when the implant was coated with the bi-functional metal-cell binding MCSP-2 peptide (Figure 8A), for a bare implant (Figure 8B) and when only the metal-binding part of the peptide, MBP-1 was used for functionalization (Figure 8C). Laminin 332 was used for immunohistochemical staining that evidenced the epithelial adhesion on the implant surface (see Supplementary Information). One can identify from the figures the main structural components, which are the connective tissue (CT), the oral epithelium (OE) and the epithelial junction (JE). The results demonstrated that the epithelial cell migration apically was successfully limited on the MCSP-2 functionalized implants (see arrows pointing to the JE-dark brown line in Figure 8A).

Apical epithelial migration on bare implant surfaces was obvious, creating a longer junctional epithelium (Figure 8B), compared to implants functionalized with the MCSP-2 bifunctional peptide where the epithelial–implant interface was much shorter (Figure 8A). It also demonstrated organized gingival conjunctive fibers (indicated by small arrows in the JE domain) as is usually observed around teeth. There was a statistically significant difference in epithelial attachment, measured as the length of JE, between the bare and the MCSP-2 functionalized implants (Figure 8D). Implants coated with the metal binding peptide MBP-1 were also included in the study (Figure 8C). In this case, the length of JE indicating epithelial adhesion was statistically different from that obtained for the bare implant, but the apical migration of the epithelial cells was not as limited as on the surfaces coated with MCSP-2.

It is evident that the proposed MCSP-2 bifunctional peptide gave the best results between compared surfaces by both increasing the gingival adhesion on the surface and successfully inhibiting the epithelial cell migration toward the apical implant part.

## 4 Discussion

The strategy to modify the Ti and Ti6Al4V substrates adopted in the present study was to use bi-functional peptides to mimic the physiological epithelial cell attachment on the normal tooth via internal basal lamina (IBL). The IBL differs significantly from a typical basement membrane in terms of its protein composition including laminin 332 (Larjava et al, 2011). Once epithelial cells have migrated to the surface of an implant, they adhere directly via basal lamina (Rompen, 2012).

Laminin-332 and E-cadherin specific sequences for designing biomimetic peptides were chosen that were able to form strong and stable adhesions with keratinocyte cells. To anchor these cell-specific peptides to Ti or Ti6Al4V 12-mer metal binding sequences were elaborated via phage display technology (Seker and Demir, 2011; Estephan et al, 2012). Amongst the four bi-functional peptides proposed in this work, only the combination resulting in MCSP-2, composed of the MBP-1 metal binding peptide and the cadherin derived peptide CSP-2, provided a favorable spatial configuration to allow double adhesion function to both Ti based metals and oral epithelial cells.

It was demonstrated that indeed the SVSVGMKPSPRP sequence (MBP-1) adhered strongly to both implant surfaces and was resistant to hydrophobic, hydrophilic and ionic rinsing procedures (see Supplementary Information). The cell binding part, H-SWELYYPLRANL-NH2 (CSP-2) was described by Devemy and Blaschuk, (2009) as the specific binding peptide of the E-cadherin ectodomain with a high binding affinity of 9.4 mM.

Both the cell culture tests and single force spectroscopy results demonstrated that the bi-functional MCSP-2 peptide increased oral keratinocyte cell adhesion on Ti and Ti6Al4V surfaces. The MCSP-2 peptide generated cell adhesion stable 4 hours after incubation even after BSA adsorption.

A similar approach using a bi-functional peptide was reported for triggering the endothelization of Ti6Al4V in endovascular prostheses, showing the relevance of the peptide route functionalization (Meyers et al, 2011). Yazici et al, (2013) used Cell surface display to select peptides with higher affinity to Ti surfaces and then a bi functional peptide was designed by combining this later peptide with an integrin recognizing peptide motif. Results showed that the functionalization of the Ti surface by the selected bi-peptides significantly enhanced the bioactivity of osteoblast and fibroblast cells on implant-grade materials. It was also demonstrated that the use of combined titanium-hydroxyapatite peptides based on titanium peptide binder (KKLPDA) and hydroxyapatite peptide binder (EEEEEEEE) can be successfully adsorbed onto Ti6Al4V and hydroxyapatite surfaces (Rodriguez et al, 2017). An Alternative way is also reported, it consists of treating the Ti6Al4V by activated vapor salinization and then covalent attaching the RGD oligopeptides to the surface to increase the attachment, spreading and rearrangement of mesenchymal stem and progenitor cells (Álvarez-López et al, 2022). Just to note that all these reports lack *in vivo* studies.

*In vivo* animal studies confirmed the capacity of our bi-functional peptide to ensure stable cell adhesion on the trans-gingival part of the dental implant and also to arrest the unwanted apical migration of the epithelial cells pointing toward promising medical applications of the proposed novel peptide. The value of the rat model confirmation of the effectiveness of the MCSP-2 bi functional peptide lies in the fact that the *in vivo* study indirectly reflects the problem with oral biofilm formation. It is known that epithelial adhesion to implant surfaces is poorer (Fujiseki et al, 2003) and therefore conducive to clinical problems such as peri-implantitis (Koldsland et al, 2010). From the results, it can be assumed that the MCSP-2 bi functional peptide by enhancing epithelial attachment arrested biofilm plaque formation and apical migration.

We believe thatthe results of the present study provide serious evidence on molecular, cellular and animal level that coating Ti implants with such bioengineered peptide represents an effective method to improve mucosal adhesion on dental implants and to reduce peri-implantitis and mucositis prevalence. The method could rapidly be clinically evaluated before finding clinical applications.

## 5 Conclusion

In order to improve osteo-integration, biocompatibility and bioactivity of titanium implant and its alloys, numerous strategies for **s**urface modifications are already known. In this work, we report on combining two peptides: one specific and selective for Ti and Ti6Al4V surfaces extracted by phage display technology and another one that can present affinity to epithelial cells. This latter bi peptide can result in powerful bioengineered peptides. Four bi functional peptides were synthesized and evaluated using single-cell force spectroscopy, surface roughness and cell viability tests. It was shown that MCSP-2 is the most effective one. Our MCSP-2 showed on cellular and animal levels its capacity to improve gingival adhesion and to diminish peri-implantitis and mucositis prevalence. This latter bi-peptide can be clinically evaluated to introduce it in the medical field where Ti and its alloys are used. i.e., manufacturing of hip, artificial knee joints and dental implant prostheses components.

## Data Availability

The datasets presented in this study can be found in online repositories. The names of the repository/repositories and accession number(s) can be found in the article/Supplementary Material.
