# Supplementary material for: Improving dental epithelial junction on dental implants with bioengineered peptides
Source: Front Bioeng Biotechnol. 2023 Jun 20;11:1165853. doi: 10.3389/fbioe.2023.1165853 (PMC10318435; doi:10.3389/fbioe.2023.1165853)
Supplement: Supplementary file 1 [file DataSheet1.docx]

**Improving dental epithelial junction on dental implants with bioengineered peptides**

Ivan V. Panayotov^1,2^, Attila G. Végh^3^, Marta Martin^4^, Boyan Vladimirov^5^, Christian Larroque^6^, Csilla Gergely^4^, Frédéric J.G. Cuisinier^1,2^, Elias Estephan^1,7*^

^1^LBN, Univ Montpellier, Montpellier, France

^2^CSERD, CHU Montpellier, Montpellier, France.

^3^Institute of Biophysics, Biological Research Centre, Eötvös Lóránd Research Network (ELKH), Szeged, Hungary.

^4^L2C, Univ Montpellier, CNRS, Montpellier, France.

^5^Department of Maxillofacial Surgery, Medical University of Plovdiv, Bulgaria.

^6^Department of Nephrology, CHU Montpellier, Hôpital Lapeyronie, IRMB, University of Montpellier, INSERM U1183, Montpellier, France.

^7^Neuroscience Research Center, Faculty of Medical Sciences, Lebanese University, Beirut, Lebanon.

*Corresponding author

**Elias Estephan, PhD.**

Associate Professor

Bioengineering Nanoscience Laboratory UR_UM104

Montpellier University

545 Avenue prof. Viala

34193 Montpellier Cedex France

Mobile : +33 7 67 64 23 75

Email : [elias.estephan@umontpellier.fr](mailto:elias.estephan@umontpellier.fr)

1 Selection of metal binding peptides (MBP) via phage display technology:

After the third biopanning round, the peptide sequence SVSVGMKPSPRP (MBP-1) was expressed by 37.5% of the phages (table 1). MBP-1 has great potential as a linker for functionalizing metallic surfaces if specificity is not a key factor [1]. To select a second peptide, 6 bacteriophage clones were used in a fourth biopanning round (each clone was amplified separately, and a bank of 6 bacteriophages was then created by mixing the clones). After this fourth round, the sequence WDPPTLKRPVSP (MBP-2) was expressed by 40% of the phages. Therefore, MBP-1 and MBP-2 were chosen as the metal binding parts of the bi-functional peptides.

| Peptides | Frequency  (round 3) | Frequency  (round 4) |
| --- | --- | --- |
| SVSVGMKPSPRP | 37.5% | 20% |
| WDPPTLKRPVSP | 12.5% | 40% |
| LPSHHTPKWGLS | 12.5% | 20% |
| GTLGGDYMKYLS | 12.5% | 20% |
| LPIDHAATHESR | 12.5% | 0% |
| HPAWALGKLNVE | 12.5% | 0% |

Supplementary Table 1: Peptide sequences and their apparition frequency as isolated after third and fourth biopanning rounds of the phage display on Ti6Al4V

The affinity of both MBPs to Ti and Ti6Al4V was tested by mass spectrometry. After adsorption of MBP-1 or MBP-2 peptides on Ti and Ti6Al4V, we evaluated their resistance to rinsing using hydrophilic, hydrophobic and ionic solutions. After rinsing, the peptides remaining on the surfaces were identified using a MALDI TOF/TOF spectrometer (Figure in the Main manuscript). The obtained mass-to-charge ratio (m/z) agreed with the theoretical mass (table 2)

| Peptide | MW ^a)^ | pI ^b)^ | Charge | II ^c)^ | AI ^d)^ | Hydropathicity ^e)^ |
| --- | --- | --- | --- | --- | --- | --- |
| MBP-1 | 1240.66 | 11 | +2 | 58.24 | 48.33 | -0.475 |
| MBP-2 | 1392.6 | 8.75 | +1 | 114.03 | 56.57 | -1.058 |

Supplementary Table 2: Physico-chemical characteristics, calculated on http://expasy.org, of the metal binding peptides

a) MW = the theoretical monoisotopic molecular weights; b) pI = the isoelectric point; c) II = the instability index of a peptide: II < 40 predicts a stable peptide, and II > 40 predicts an unstable peptide; d) AI = the aliphatic index; e) Hydropathicity, calculated as the sum of the hydropathist of the individual amino acids divided by the number of residues in the sequence. Larger values indicate more hydrophobic amino acids or peptides.

MBP-1 peptide was identified on both Ti and Ti_6_Al_4_V surfaces after the three types of rinsing (table 3).

|  | MBP-1 | | | MBP-2 | | |
| --- | --- | --- | --- | --- | --- | --- |
|  | Acetonitrile | H_2_O | 1 M NaCl | Acetonitrile | H_2_O | 1 M NaCl |
| Ti | detected | detected | detected | detected | Not - detected | Not - detected |
| Ti_6_Al_4_V | detected | detected | detected | detected | Not - detected | Not - detected |

Supplementary Table 3: MALDI/TOF-TOF detection of MBP-1 and MBP-2 peptides on Ti and Ti6Al4V surfaces after rinsing with acetonitrile, water or 1 M NaCl.

The sequence of the MBP-1 peptide presents a hydrophobic first half and a hydrophilic second half. The fact that the peptide remains on the surface after acetonitrile rinsing, which is known to break hydrophobic links, suggests a second alternative binding mechanism that is most likely electrostatic in nature. However, the adhesion is also resistant to a high ionic strength solvent, indicating that electrostatic interactions are not critical. The existence of two complementary adhesion mechanisms in a 12-mer peptide is surprising, but it explains the resistance to rinsing and the ubiquity of adherence of this peptide. This is also the reason why the phage was considered to be a “superinfectious” phage present in the New England Biolabs Library (New England Biolabs, Ipswich, MA, USA) due to a high amplification potential and its resistance to rinsing[2]. The second metal binding peptide (MBP-2) was not detected after rinsing with hydrophilic (water) or ionic solutions (1 M NaCl). The MBP-2 peptide contains two positively charged amino acids (K and R) and one negatively charged amino acid (D); its total charge is thus twofold lower than the charge of MBP-1 (table VIII). This is most likely the reason for its lower electrostatic interaction with the negatively charged metal surfaces, as indicated by its lack of resistance to rinsing with 1 M NaCl. The mass–spectrometry spectra of MBP-2 (Figures 1C and 1D in the main text) reveal two peaks at m/z 1406.6 and m/z 1423.6, suggesting the presence of oxygen ions in the peptide structure. It is to note that in ten sequences with high affinity to Ti6Al4V, the amino acids D, C and S occur most frequently[3]. Previously, the general oxide binding motifs –SPS- and –SGS- were proposed using molecular modeling[4]. Comparing our MBP-1 and MBP-2 sequences, it is difficult to arrive at a general motif for metal oxide adhesion.

2 Detailed methods:

***2.1 Single-cell force spectroscopy (SCFS):***

Tip-less cantilevers (MikroMasch, Tallinn, Estonia) were used for cell-implant surface adhesion measurements. (see Supplementary Figure 1).





Supplementary Figure 1: Typical unbinding of a single keratinocyte from the Ti surface measured using AFM.

The loading rate was set to 2 µm/s. After reaching the maximal loading force, the contact was maintained for 5 s (at a constant piezo z position). Small adhesion events were recorded in the retracting curve (black curve), even at long pulling distances. Some of these events were preceded by a force plateau that could suggest membrane tethers (arrows). These small events were not considered in our analysis. The inset shows an image of a keratinocyte cell adsorbed on the tip-less AFM cantilever. Images of the cantilever were recorded before and after each set of measurements and compared. No significant difference was observed among the images. Scale bar represents 18 μm.

*2.2 In vivo* rat experiments

2.2.1 Implants

The screw-type implants used in this study were fabricated from Ti6Al4V grade IV (Kondo Technology, Japan; Fig. 1a and b). Before use, the implants were cleaned in sodium dodecyl sulfate 0.1 M (Sigma Aldrich, St. Louis, USA), in hydrochloric acid 0.1% (Sigma Aldrich, St. Louis, US) and finally in ultra-clean water (Milli-Q; Merck Millipore, Darmstadt, Germany) at 22°C in an ultrasound bath for 5 min. Samples were individually autoclaved at pressure of 1bar and 121°C for 20 min.

2.2.2 Oral implantation

The oral implantation procedure was completed according to the immediate-implantation model, as described from Ikeda at al. (2000) [5]. Briefly, 12-week-old Wistar rats (male, 300–320 g) were anesthetized with intraperitoneal pentobarbital sodium (50 mg/ kg; ref), and the first right maxillary molar was extracted. Half the animals received implants were inserted immediately into the prepared bone cavity. Implanted animals were sacrificed 4 weeks after implantation surgery (see Supplementary Figure 2).


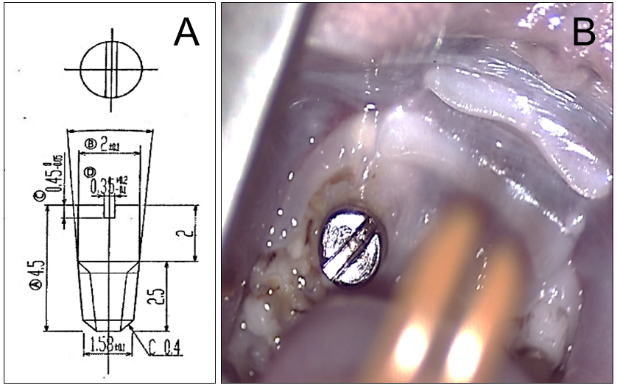


Supplementary Figure 2: (A) design of the implant; (B) 4 weeks after implantation

References:

[1] E. Estephan *et al.*, *Biomed Tech Berl*, vol. 57, p. 481, 2012.

[2] G. Kolb, C. Boiziau, and R. N. A. Biol, vol. 2. p. 28, 2005.

[3] S. R. H. Meyers, W. P.T., K. E.B., G. D.J., and A. M. M.W., p. 2492, 2007.

[4] B. H. Lower, R. D. Lins, Z. Oestreicher, T. P. Straatsma, L. S. M. F. Hochella Jr., and S. K. Lower, *Environ. Sci. Technol.*, vol. 42, p. 3821, 2008.

[5] H. Ikeda *et al.*, *J. Periodontol.*, vol. 71, p. 961, 2000.
